# The Impact of Teachers’ Innovative Support Behaviors on Creative Anxiety Among Art and Design Majors in the Context of Innovation Education: The Mediating Roles of Creative Self-Efficacy and Achievement Motivation

**DOI:** 10.3390/bs16030336

**Published:** 2026-02-27

**Authors:** He Huang, Heung Kou

**Affiliations:** Graduate School of Education, Sehan University, Yeongam-gun 58447, Jeollanam-do, Republic of Korea; huang@office.sehan.ac.kr

**Keywords:** innovative support behaviors, creative anxiety, creative self-efficacy, achievement motivation, art and design education

## Abstract

In the age of innovation, the mission of higher education is to cultivate innovative talent. Teachers’ innovative support behaviors have a significant impact on students’ emotions and creative performance. As students at art and design colleges will generally confront creative anxiety—an unfavorable emotion that affects their innovative abilities—this study aims to explore the mediating effects of creative self-efficacy and achievement motivation in the relationship between teachers’ innovative support behaviors and the creative anxiety of art and design students. Based on random cluster sampling, a questionnaire survey was used to collect 785 valid questionnaires from undergraduate art and design students attending six universities across eastern, central, and western China for the analysis of four variables: teachers’ innovative support behaviors, creative self-efficacy, achievement motivation, and creative anxiety. The results show that (1) teachers’ innovative support behaviors significantly negatively predict students’ creative anxiety (β = −0.631, *p* < 0.001); (2) creative self-efficacy and achievement motivation both have significant mediating effects (β = −0.241, *p* < 0.001; β = −0.183, *p* < 0.05, respectively); and (3) these two factors present a chain mediation effect. Bootstrap tests showed an indirect effect of −0.062 with a 95% confidence interval [−0.121, −0.008]; as the interval did not include zero, this chained mediating effect was significant. The results indicate that teachers’ innovative support behaviors not only have a direct effect on alleviating students’ anxiety but also have indirect effects through motivational and cognitive mechanisms, which provides a new theoretical reference with practical significance for art and design education.

## 1. Introduction

The 21st century is characterized by greater competition in global economic and cultural spheres, which have become the focal points of nations as driving engines of national development. It is against this backdrop that the education sector has also been charged with the significant role of serving as a cradle in which innovative talent is nurtured, whereby higher education institutions have come to be considered as the pillars of national innovation systems. In recent years, the Chinese government has unveiled a number of strategic policy documents that are geared towards empowering the innovative role of higher education. A new wave of educational reforms, which began as the Project of Improving the Quality and Reforms of Teaching in Higher Education Institutions, was opened in 2008. The National Medium- and Long-term Education Reform and Development Plan (2010–2020), which was released in 2010, expressly noted that to develop an innovative nation, education should be given the highest priority (Ministry of Education of the People’s Republic of China, 2010). Further policies, such as the Double First-Class Initiative, the Declaration on the Development of the New Liberal Arts, and the Undergraduate Teaching Quality Enhancement Project, have led to fundamental changes in the most significant aspects of the process of university education, specifically, curriculum, teaching process, and evaluation systems (State Council of the People’s Republic of China, 2015; Ministry of Education of the People’s Republic of China, 2021). In the framework of such innovation-oriented policies, the very notion of innovation in Chinese higher education—especially in the fields of art and design—is explained as the ability to produce new and productive ideas, solutions, or pieces of art that do not adhere to the rules. It reaches beyond technical aptitude to include critical thinking, problem-solving, and synthesizing knowledge across domains, thereby supporting the national strategic objective of developing talent to work in the cultural and creative sectors (Ministry of Education of the People’s Republic of China, 2021). As a consequence, the roles of university faculty have changed significantly. The information presented in the report Teachers as Designers of Learning Environments by the Organisation for Economic Co-Operation and Development (OECD) focuses on the fact that modern educators should not only be innovative in academic research, but must also demonstrate professional creativity in instructional design, pedagogical innovation, and development of the classroom environment (OECD, 2019). Teachers must play the role of catalyzers for the innovative thinking of students, guide them during the innovation process, and nurture an innovative culture among students. This is especially the case in creative fields such as art and design, where the innovative support behaviors of teachers play a central role in the innovative performance of students.

### 1.1. Creative Anxiety: Emotional Barriers in Innovation Education

The learning experience for art and design majors is creative, uncertain, and exploratory (Green et al., 2024). Unlike other fields, where students are taught correct answers, in art and design education students are taught to challenge the status quo, to venture into the unknown, and to be themselves—a learning process that is often highly psychologically challenging (Green et al., 2024).

When faced with open-ended and difficult innovation tasks, students are likely to encounter “creative anxiety,” which refers to a cluster of emotional responses manifested as worry, unease, and tension in response to concerns about the possible negative consequences of novelty, evaluation, failure, and uncertainty during creative activities (Kapoor, 2024; Zhou et al., 2023).

As a distinct emotional state, creative anxiety is salient and present in innovation education settings. Similarly to other domain-specific anxieties, such as test anxiety and mathematics anxiety, creative anxiety can affect students’ learning process and creative work. Experimental studies have shown that students with high levels of creative anxiety present hindered performance in divergent thinking, metaphorical transfer, and hypothesis formation, which in turn leads to a reduction in both the quantity and quality of creativity (Daker et al., 2023).

At present, creative anxiety is a significant but limited area of focus in the existing body of research on art and design education. Regarding positive emotions related to the creative experience—for example, relative to creative enjoyment and flow experiences—the literature is rich; in contrast, studies focusing on negative emotions, particularly creative anxiety, are relatively scarce (Akpur, 2024; Hoffmann et al., 2021; Zhou et al., 2023). Therefore, the determinants and mitigation of creative anxiety in art and design students hold both theoretical and applied importance.

### 1.2. The Key Role of Teachers’ Innovative Support Behaviors

The adaptive support behaviors of teachers in higher education contexts are seen as one of the contextual determinants that affect the innovative performance and emotional experiences of students. Innovative support behaviors are a group of instructional behaviors referring to the ways in which teachers act to create supportive environments (e.g., through course design and pedagogical activities) to support innovative activities among students. Such practices include supplying resources and tools to promote innovation, giving room for autonomous exploration, giving feedback in a timely and effective manner, fostering psychological safety, and embracing failures and mistakes (Beghetto, 2021; Li & Liu, 2023; Wu et al., 2022).

Regarding Resource Conservation Theory (Bakker & Demerouti, 2024; Demerouti & Bakker, 2023; Hobfoll, 1989), students receive essential resources in the form of the innovative support behaviors of teachers. When faced with difficult innovative tasks, the instrumental, informational, and affective assistance provided by teachers can help students to better cope with the demands of the task, diminish the possibility of resource drainage and, thus, minimize anxiety experiences. In art and design projects especially, where students may face various material limitations, technical problems, and creative bottlenecks, the professional advice and assistance of the teacher significantly contribute to feelings of resource security in the student.

Regarding Self-Determination Theory (Ryan et al., 2021; Vansteenkiste et al., 2023), the innovative support behaviors of the teachers should promote the psychological wellness of students, thus satisfying their primary psychological needs related to autonomy, competence, and relatedness. The level of intrinsic motivation in students is improved when they are given the room to explore freely; in this respect, teachers can offer them aid to help them to develop skills and create a positive teacher–student relationship, leading to improved emotional regulation. This also provides relief against the anxiety caused by creative activities.

Furthermore, based on Social Cognitive Theory (Albarracín et al., 2024; Bandura, 1997; Deng et al., 2025), the innovative support actions of teachers control fear reactions as they influence the process of cognitive appraisal in students. When the support of teachers is perceived, the students may find innovative tasks challenging but not threatening; such a positive cognitive reaction will then be converted into adaptive emotional reactions. Empirical studies offer supportive evidence for the association between the innovative support practices of teachers and the emotional wellbeing of students. Zou et al. (2024) reported that the emotional support of teachers positively increased students’ intrinsic motivation by decreasing anxiety and increasing their efficacy. Likewise, Liu et al. (2021) showed that perceived teacher support is positively related to both divergent and insightful thinking in students, with creative self-efficacy acting as a mediator. As these studies did not study creative anxiety per se, they provide only indirect evidence on the ways in which the innovative support behaviors of teachers affect student wellbeing.

These three theories, as a cohesive system, define how creative anxiety can be mitigated through the innovative support behaviors of teachers. In particular, COR represents the need to provide external resources to reduce perceived threats; SDT underlines the need to satisfy psychological wellbeing to intrinsically motivate students; and SCT places emphasis on cognitive evaluation of the emotional reaction to reshape it. This multi-theoretical combination offers an inclusive prism through which we may analyze the interactions between support and student emotions in creative disciplines occurring via environmental, motivational, and cognitive mechanisms.

### 1.3. The Mediating Role of Creative Self-Efficacy

Creative self-efficacy (CSE) is the belief of an individual regarding their capability to generate, apply, and optimize creative ideas; i.e., their belief in their ability to accomplish creative tasks successfully (Kwon & Kim, 2020; Yin et al., 2024). Self-efficacy theory posits that self-efficacy is a significant psychological determinant in situations where individuals face difficult tasks, with its application affecting their motivation, intensity of effort, persistence, and emotional experience (Bandura, 1997). In the literature on creativity, creative self-efficacy is considered a very important mediating factor between environmental variables; for example, between the innovative support behaviors of teachers and creative performance.

Broadly, the existing literature suggests that creative self-efficacy is a behavior of students that is strongly predicted by innovative support practices in teachers. As an example, Shi et al. (2023) established a positive significant association between perceived teacher support and creative self-efficacy, indicating that greater perceived teacher support results in greater self-belief regarding creative potential in students. On the same note, Zhang et al. (2022) noted that the relationship between instructional support and creative output is mediated by creative self-efficacy.

In the light of emotional regulation, self-efficacy is an imperative buffering aspect that influences anxiety experiences. Students with high creative self-efficacy tend to view innovation tasks as challenges, and not threats that can hardly be controlled. The outcome of this positive cognitive appraisal is fewer episodes of anxiety (Hasani, 2024). On the other hand, students with low self-efficacy tend to overestimate the difficulty of tasks, underestimate their coping skills, and are more susceptible to anxiety reactions (Hascher & Waber, 2021; Pekrun, 2024).

Creative self-efficacy can play a very significant role, especially in the context of art and design education. Continuous problem-solving and the need to overcome whatever problems arise are inherent to the creative process, and a student’s confidence in their creative abilities directly determines how they react to problems, especially regarding emotions. When taught to possess stronger creative self-efficacy through means of assistance including scaffolding support, successful experiences, and positive student feedback, creative anxiety is reduced as students take a more positive approach when encountering creative uncertainties and challenges.

### 1.4. The Mediating Role of Achievement Motivation

Achievement motivation refers the internal motivation that gives people the drive to work towards success, cope with obstacles, and reach high levels of excellence (Brunstein & Heckhausen, 2018). Classical theories of achievement motivation imply that people with higher achievement motivation levels are associated with the choice of moderately challenging tasks, favorable work conditions in which clear feedback is obtained, and greater persistence when confronted with challenges (Elliot & McGregor, 2001). In the setting of innovation education, achievement motivation serves as a primary mechanism driving students towards engaging in innovative activities.

The innovative support behaviors of teachers can have various effects on the achievement motivation of students. To begin with, teachers can foster the development of clear goal expectations among students through setting moderately challenging tasks, clearly stating the evaluation criteria, and providing feedback to students in a timely manner, thus inciting their desire to attain success (Liu et al., 2021). Second, educators can promote the psychological safety of students by making the classroom an encouraging and welcoming environment, which can help students to feel comfortable in taking on challenging activities without feeling too worried about possible failure (Ryan et al., 2021; Vansteenkiste et al., 2023).

Achievement motivation plays an influential role in creative anxiety, which can be explained in terms of the following mechanisms. High achievement-motivated students are more at ease with task-oriented coping, which allows them to focus on problem-solving instead of emotional distress (Pekrun, 2024). Creative challenges or disappointments do not stop them because of their desire to achieve success; as such, they strive to find out what they can do to rectify the situation instead of being overwhelmed with anxiety. Moreover, more achievement-oriented people normally have more confidence in their skills, which, in effect, acts as a buffer against anxiety caused by external demands (Elliot & McGregor, 2001).

### 1.5. The Chain Mediating Effect: A Theoretical Framework

This study further suggests that achievement motivation and creative self-efficacy form a chain of mediation that can be used to jointly describe the relationship between the innovative support behaviors of teachers and the creative anxiety of students. This assumption relates to the fact that environmental influences (e.g., teacher support) contribute to the motivation orientations (e.g., achievement motivation) of individuals; therefore, such influence will have an impact on their cognitive appraisals (e.g., creative self-efficacy) and, ultimately, control their emotional reactions (e.g., creative anxiety). This model of chain mediation is therefore a dynamic process at the psychological level, whereby motivation promotes thought which, in turn, influences emotion.

First, teachers’ innovative support may positively affect students’ achievement motivation. When students perceive an innovative supportive instructional environment, their achievement motivation (i.e., their willingness to put forward effort to achieve goals) increases; that is, they become more willing to engage in innovative tasks (Brunstein & Heckhausen, 2018). This increase in achievement motivation then positively affects students’ engagement in creative tasks. When students engage in such tasks and have successful experiences, their creative self-efficacy is positively affected (Liu et al., 2021).

Then, when students have high levels of creative self-efficacy, they feel that they have control over creative tasks and, hence, feel less threatened and uncertain; that is, their creative anxiety decreases. This chain mediation model integrates achievement motivation theory and cognitive appraisal theory, thus explaining the dynamic psychological processes underlying the relationship between environmental support and emotion. Previous studies have investigated the relationships among teacher support, self-efficacy, motivation, and anxiety separately, but few have explored the sequential mediating effects of achievement motivation and creative self-efficacy on the relationships between teacher support and anxiety in the context of art and design education.

## 2. Research Hypotheses

On the theoretical basis mentioned above, this research attempts to answer the following research questions: (1) What is the extent to which the innovative support behaviors of teachers directly reduce creative anxiety in art and design students? (2) Does creative self-efficacy have a mediating effect in this relationship? (3) Is achievement motivation also a mediating variable? (4) Is there a chain mediation pathway for the relationship between teachers’ innovative support behaviors and creative anxiety involving achievement motivation and creative self-efficacy?

**Figure 1:**
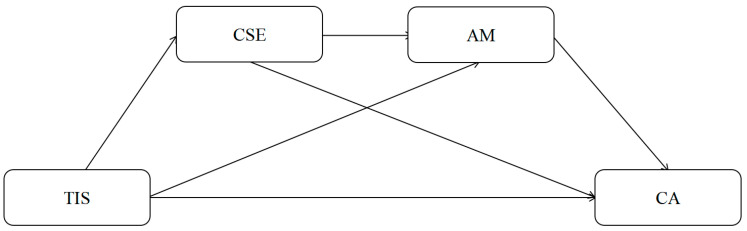
Hypothetical model diagram. *Note*: TIS, teachers’ innovative support; AM, achievement motivation; CSE, creative self-efficacy; CA, creative anxiety.

To answer these questions, the following hypotheses and the conceptual model shown in Figure 1 were developed.

**H1.**  *There is a negative and significant relationship between innovative support-related behaviors of teachers and creative anxiety in art and design university students.*

**H2.**  *Creative self-efficacy mediates the relationship between the innovative support behaviors of teachers and creative anxiety.*

**H3.**  *Achievement motivation mediates the relationship between the innovative support behaviors of teachers and creative anxiety.*

**H4.**  *A mediation chain involving creative self-efficacy and achievement motivation exists in the relationship between teachers’ innovative support behaviors and creative anxiety. In particular, the innovative support behaviors of teachers contribute to the achievement motivation of students, which eventually boosts their creative self-efficacy, resulting in a lower degree of creative anxiety in the long-term*.

## 3. Research Methodology

### 3.1. Research Design and Methodology Overview

The study follows a cross-sectional survey design, with data collection carried out using a questionnaire to investigate the mechanism of influence between teachers’ innovative support behaviors and creative anxiety in art and design students. According to the literature analysis and theoretical considerations, we utilized a serial mediation model to test the sequential mediating effects of creative self-efficacy and achievement motivation. The research was conducted on the basis of a typical methodology for a psychological quantitative study, entailing the choice of instruments, incorporation of sampling, data collection, and statistical analysis (Durlak, 2009; Podsakoff et al., 2012).

We adopted a cluster sampling method based on the principle of convenience to ensure methodological representativeness of the sample, and utilized standardized measurement tools with appropriate reliability and efficacy to manage measurement errors. The statistical tools used to analyze the data included correlation analysis, regression analysis, and bootstrap mediation effect tests, enabling comprehensive testing of the research hypotheses. In the sections below, the traits of the study participants, the psychometric characteristics of the research tools, and the precise data analysis methodology are detailed.

### 3.2. Research Participants

In this research, a random cluster sampling technique was used. Between September 2023 and January 2024, six universities in eastern, central, and western China were sampled to recruit full-time undergraduate art and design majors; in particular, two of the universities were in the Double First-Class category while the other four were ordinary undergraduate establishments. The chosen institutions included total, art-oriented, and teacher-training universities to ensure the representativeness of the sample. The fields comprised a wide range of disciplines, including visual communication design, environmental design, product design, and digital media arts.

The researchers initially contacted the heads of the art departments in each university to introduce the aims and methods of the study. After it was agreed upon, the questionnaires were distributed to the existing class groups as a unit. A total of 850 questionnaires were issued. After filtering questionnaires presenting invalid responses, extreme repetitive patterns, filled out within a very short time, and missing data, 785 valid questionnaires were retained to be analyzed, resulting in a response rate of 92.4%.

The sample consisted of 284 male (36.2) and 501 female (63.8) students aged 17–24 (M = 20.12, SD = 1.43). The grade distribution was as follows: 218 first-year, 245 second-year, 197 third-year, and 125 fourth-year students (27.8%, 31.2%, 25.1%, and 15.9%, respectively). Informed consent was given by all participants, and it was guaranteed that all data were to be kept confidential and utilized solely in an academic context. The research protocol received the consent of the Zhengzes University Ethics Committee (ZDLL-20230720), and was carried out in compliance with the principles of the Ethical Principles of the Declaration of Helsinki of 1964 and its future amendments. 

### 3.3. Research Tools

#### 3.3.1. Teacher Innovation Support Behavior Scale

In the given study, the Chinese version of the Innovation Support Behavior Scale created by Zhou and George (2001) was applied. The scale has found extensive use in studying organizational behaviors and, in this study, its use was modified to fit the context of art and design courses; in particular, all questions started with the stem “In my art and design courses, my teachers …” The initial scale comprised 11 items that cover the dimensions of the provision of creative resources, recognition of innovations, and permission to make independent decisions. An example statement is “My instructor is willing to experiment with new and non-traditional approaches to design, and when we suggest something new, the instructor offers us positive feedback”. The reliability analysis revealed a high internal consistency coefficient (Cronbach’s α = 0.93), indicating high reliability. The confirmatory factor analysis using maximum likelihood estimation showed a good fit to the model (χ^2^/df = 2.85, CFI = 0.96, TLI = 0.95, RMSEA = 0.06) and acceptable construct validity. The normality of all scale items was assessed before CFA. The results showed that the skewness of the items had an absolute value ranging from 0.12 to 0.35, while the kurtosis had an absolute value ranging from 0.08 to 0.42. The assumptions of normality were satisfied in all cases (Kline, 2023).

#### 3.3.2. Creative Self-Efficacy Scale

This study further employed the Chinese version of the Creative Self-Efficacy Scale originally developed by Tierney and Farmer (2002) and subsequently adapted for the local context. This scale comprises three items that measure individuals’ beliefs about their ability to perform creative tasks. Individuals were asked to agree or disagree with each statement in the context of studying art and design; examples of statements include “I believe I have the ability to propose creative solutions in design projects” and “I am good at creative design compared with my peers”. Although the scale is short, it has been extensively used in creativity studies due to its well-defined conceptual clarity. In the present study, the scale also demonstrated high internal consistency (Cronbach’s α = 0.87). The confirmatory factor analysis using maximum likelihood estimation showed a good fit to the model (χ^2^/df = 1.98, CFI = 0.99, TLI = 0.98, RMSEA = 0.04). Descriptive statistics were assessed for all items of the scale before carrying out the CFA. The test results showed that the absolute values of skewness for all items ranged between 0.12 and 0.35, while those for kurtosis ranged between 0.08 and 0.42. Again, all results fulfilled the assumptions of normality (Kline, 2023).

#### 3.3.3. Achievement Motivation Scale

This research used the Chinese revised version of the Achievement Motive Scale formulated by Yu and Yang (1994). Based on Atkinson’s achievement motivation theory, the scale distinguishes between two factors: motivation to succeed (MS) and motivation to avoid failure (MF). For this study, only the MS subscale (8 items) was used to measure achievement motivation. This decision was grounded in the theoretical focus on proactive, approach-oriented motivation in innovation education (Beghetto, 2021), which aligns with the MS subscale’s emphasis on pursuing success and mastering challenges. In contrast, the MF subscale, which focuses on avoidance behaviors, was excluded to avoid conceptual overlap with creative anxiety and to maintain clarity in modeling adaptive motivational processes. The MS subscale showed high internal consistency (Cronbach’s α = 0.90) in this study. The confirmatory factor analysis using maximum likelihood estimation showed a good fit to the model (χ^2^/df = 2.41, CFI = 0.95, TLI = 0.93, RMSEA = 0.05), indicating its structural validity. The CFA was conducted after applying normality tests to all the scale items. The absolute values of skewness for individual items were in the range of 0.12 to 0.35, whereas those for kurtosis were in the range of 0.08 to 0.42. All outcomes satisfied the assumptions of normality (Kline, 2023).

#### 3.3.4. Creative Anxiety Scale

In this study, a subscale of the Creative Achievement Emotion Questionnaire (CAEQ) known as the creative anxiety subscale, which was first created and developed by Kaufman and Beghetto (2018), was employed. The subscale is composed of five questions designed to evaluate the negative emotions (e.g., tension and worry) experienced by people performing creative tasks (e.g., artistic design). Example statements include “I usually get nervous when I have a new design project” and “I fear that my piece of work is not creative enough and can disappoint my teacher”. The researchers translated and back-translated the Chinese version that was used in this study, which was later checked by experts to ensure semantic equivalence. Evidence of good internal consistency of the subscale was obtained in the present study (Cronbach’s α = 0.88). The confirmatory factor analysis using maximum likelihood estimation showed a good fit to the model (χ^2^/df = 2.67, CFI = 0.97, TLI = 0.95, RMSEA = 0.05), with the reliability and validity both within accepted psychometric standards. The normality of all scale items was assessed before undertaking the CFA. The absolute values of skewness for all items were between 0.12 and 0.35, while those for kurtosis were between 0.08 and 0.42. The assumption of normality was satisfied in all cases (Kline, 2023).

### 3.4. Data Statistics and Analysis

The study employed SPSS 26.0 for descriptive statistics, correlation analysis, and common method bias testing. The moderated mediation model was validated using the SPSS macro Process Model 6.

## 4. Results

### 4.1. Common Method Bias Test

As the data supporting the research outcomes were gathered using self-reported questionnaires, the possibility of common method bias must be addressed. To check for the existence of common method bias, exploratory factor analysis (EFA) of all items in all scales (35 items) was carried out using Harman’s single-factor test. The estimation method used was Principal Axis Factoring, which is considered appropriate for the discovery of an underlying factor structure and has less restrictive implications with regard to the data distribution (Costello & Osborne, 2005). The outcomes demonstrated that the variance captured by the initial unrotated element was 28.7%, less than the widely recognized threshold of 40%. These findings indicate that the issue of common method bias was not a major concern in the present study.

### 4.2. Correlation Analysis

First, descriptive statistics and Pearson correlation analyses were performed for the core variables of the study—teachers’ innovative support behavior, creative self-efficacy, achievement motivation, and creative anxiety. The results are summarized in Table 1.

The results shown in Table 1 indicate that correlations between the study variables were significant. Teachers’ innovative support behavior showed significant positive correlations with creative self-efficacy (r = 0.52, *p* < 0.001) and achievement motivation (r = 0.48, *p* < 0.001), and a significant negative correlation with creative anxiety (r = −0.36, *p* < 0.001). Creative self-efficacy was positively correlated with achievement motivation (r = 0.56, *p* < 0.001), and both mediating variables were negatively correlated with creative anxiety (CSE: r = −0.45, *p* < 0.01; AM: r = −0.41, *p* < 0.001). Additionally, the internal consistency coefficients (Cronbach’s α) for all scales ranged from 0.87 to 0.93. These results provide preliminary support for the hypothesized relationships between the variables and satisfy the conditions for subsequent mediation analysis.

### 4.3. Testing the Chain Mediation Model

Before conducting mediation analysis, we performed multicollinearity tests on all predictor variables (TIS, CSE, AM) and calculated the Variance Inflation Factor (VIF). The results showed that the VIF values for all variables ranged from 1.23 to 1.87—all significantly below the critical threshold of 10 (Hair, 2009), indicating that the model does not exhibit multicollinearity issues and satisfies the statistical assumptions for mediation analysis.

The SPSS macro Process was used to examine the moderated mediation model using 5000 bootstrap samples, with Model 6 employed for validation. In particular, the moderating effect of perceived social support and the mediating effect of life meaning were explored. The specific results are as follows.

**Figure 2:**
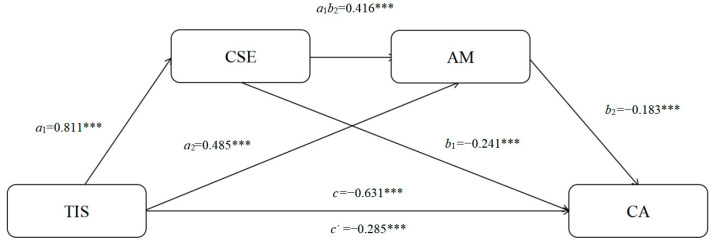
Validation model diagram. *** *p* < 0.001.

Based on the results of the chain mediation model test (Table 2 and Table 3, Figure 2), hierarchical regression analysis and the bootstrap method were employed for validation. The hierarchical regression results indicated that teachers’ innovative support behaviors significantly and negatively predicted creative anxiety (β = −0.631, *p* < 0.001) and significantly and positively predicted creative self-efficacy (β = 0.811, *p* < 0.001). When creative self-efficacy and achievement motivation were simultaneously entered into the model, teachers’ innovative support behavior (β = −0.285, *p* < 0.001), creative self-efficacy (β = −0.241, *p* < 0.001), and achievement motivation (β = −0.183, *p* < 0.05) all significantly and negatively predicted creative anxiety, thereby meeting the conditions for mediation analysis. The bootstrap results revealed a total indirect effect of −0.346, with a 95% confidence interval [−0.481, −0.210] that did not include zero. Specifically, the confidence intervals for the indirect paths “Innovative Support Behavior → Creative Self-Efficacy → Creative Anxiety” (effect = −0.196), “Innovative Support Behavior → Achievement Motivation → Creative Anxiety” (effect = −0.089), and “Innovative Support Behavior → Creative Self-Efficacy → Achievement Motivation → Creative Anxiety” (effect = −0.062) all excluded zero. These findings indicate that creative self-efficacy and achievement motivation exert both independent and sequential mediating effects in the relationship between teachers’ innovative support behaviors and creative anxiety, thereby supporting the research hypotheses.

## 5. Discussion

### 5.1. The Direct Effect of Teachers’ Innovative Support Behaviors on Creative Anxiety

This study found that teachers’ innovative support behaviors were significantly and negatively associated with creative anxiety among university students majoring in art and design. This result aligns closely with existing theoretical and empirical evidence.

First, from the perspective of Conservation of Resources (COR) theory, teachers’ innovative support behaviors help to reduce students’ risk of resource depletion when they face demanding creative tasks by providing emotional, informational, and instrumental resources (Hobfoll, 1989; Bakker & Demerouti, 2024). In art and design disciplines, students frequently engage in open-ended and uncertain creative tasks. Timely feedback, emotional care, and the provision of creative resources from teachers can mitigate students’ concerns about failure, evaluation, and uncertainty, thereby alleviating experiences of anxiety (Wu et al., 2022).

Second, based on Self-Determination Theory (SDT), teachers’ innovative support behaviors can satisfy students’ basic psychological needs for autonomy, competence, and relatedness; namely, when students believe that teachers offer them opportunities to explore independently, enhance competence, and experience positive emotions, they are more likely to perceive creative tasks as challenges rather than threats (Ryan et al., 2021; Vansteenkiste et al., 2023). Such cognitive appraisal effectively buffers students’ anxiety. Similarly, related research has also reported that the perceived teacher support not only alleviates anxiety, but can also improve students’ motivation and creative performance (Liu et al., 2021; Zou et al., 2024).

Furthermore, Social Cognitive Theory (SCT) implies that people’s emotions are mostly based on their cognitive appraisals of environmental support (Bandura, 1997; Albarracín et al., 2024). When students believe that teachers have understood and supported their exploration of creativity, they are more likely to make positive attributions and self-reflexive explanations about the uncertainty of tasks to reduce their anxiety levels. This conclusion is theoretically consistent with and validates the view of Kapoor (2024) that creative anxiety originates from uncertainty and negative expectations.

In summary, teachers’ innovative support behaviors can alleviate the anxiety that art and design students experience when performing creative tasks. This result is consistent with previous studies (Beghetto, 2021; Li & Liu, 2023), and provides empirical support for the incorporation of innovation behaviors in art and design teaching.

### 5.2. The Mediating Role of Creative Self-Efficacy

The results of this study proved that creative self-efficacy has a mediating role in the relationship between the innovation support behaviors of teachers and creative anxiety in students. In particular, the innovative support behaviors of teachers improve the creative self-efficacy of the students, thus minimizing levels of creative anxiety in students. This observation conforms to the self-efficacy theory proposed by Bandura (1997), which implies that self-efficacy is not only a determinant of the drive and persistence of individuals, but also plays a role in the emotions they develop towards the task challenge.

The association between teacher support and creative self-efficacy has also been validated in previous studies. Shi et al. (2023) found a very strong positive correlation between perceived teacher support and creative self-efficacy, indicating that the environment and sharing of knowledge could enable teachers to enhance students’ self-belief in their creative potential. Similarly, Zhang et al. (2022) discovered that creative self-efficacy acts as a very important intermediate in the relationship between teacher responsiveness and student creativity.

The present study’s results also suggest that creative self-efficacy acts as crucial psychological capital in reducing creative anxiety. Highly creative students that have high self-efficacy tend to view intricate creative activities as moderately challenging, as opposed to feeling hopelessly intimidated by them. This positive cognitive appraisal helps to reduce creative anxiety (Hasani, 2024). On the other hand, individuals with low self-efficacy tend to underestimate their skills and overestimate task complexity, leading to increased anxiety (Hascher & Waber, 2021; Pekrun, 2024) in students on creative work.

Therefore, this research confirms the positive predictive impact of the innovative facilitative behaviors of teachers on creative self-efficacy, while additionally outlining the mental process through which creative self-efficacy reduces creative anxiety.

### 5.3. The Mediating Role of Achievement Motivation

The results also suggest that the relationship between the innovative support behaviors of teachers and creative anxiety in students is mediated by achievement motivation. Put differently, the innovative assistance of teachers could help to eliminate the creative anxiety of students by increasing their achievement motivation; this outcome is in agreement with Achievement Motivation Theory (Elliot & McGregor, 2001; Brunstein & Heckhausen, 2018).

The factor of achievement motivation is particularly relevant in innovative learning settings. On one hand, an instructor can raise the standards of achievement in pupils by giving them tasks that are at the right level of difficulty and providing effective, honest feedback, thus provoking their desire to achieve excellence (Liu et al., 2021). Conversely, an encouraging and embracing classroom mindset helps students to be willing to take innovative actions due to a reduced fear of failure (Ryan et al., 2021). This motivational orientation not only increases the persistence of students, but also creates a positive orientation towards coping with uncertainty.

In line with this finding, the Control-Value Theory developed by Pekrun (2024) states that high achievement motivation implies a more cooperative attitude with leaders, thus favoring task-related coping mechanisms, placing greater emphasis on solving problems instead of emotional stress and, therefore, resulting in lower levels of anxiety. Art and design programs are processes of learning which are inherently exploratory and associated with significant creative risk. Students with high achievement motivation are reluctant to perceive failure as an adverse part of development and education, thus lessening their anxiety levels.

Therefore, the innovative support behaviors of teachers reduce creative anxiety by increasing achievement motivation in students. This result has valuable implications regarding the design of instructional interventions, which should be effectively leveraged to develop motivation and emotional resilience in students.

### 5.4. Chain Mediation: The Dynamic Mechanism of Achievement Motivation and Creative Self-Efficacy

Importantly, the results revealed that achievement motivation and creative self-efficacy have a chain mediating effect in the relationship between the innovative support behaviors of teachers and creative anxiety in students. This demonstrates that teacher support not only has a direct impact on the emotional experiences of students, but also decreases anxiety in students through a complex motivation–cognition–emotion mechanism (Bandhu et al., 2024).

In particular, the innovative support behaviors of teachers initially improve the achievement motivation of students, thus improving their readiness to perform creative activities. The higher their achievement motivation, the more likely the students are to have successful experiences in the creative process, consequently strengthening their creative self-efficacy. In turn, this increased self-efficacy further reduces the anxiety experienced by students when tackling complex and unpredictable creative tasks.

The study not only contributes to the existing body of research by confirming the importance of innovative teacher support, achievement motivation, self-efficacy, and emotion individually, but it also incorporates them into a complete chain mediation model in the context of art and design education. This framework highlights the interactive processes of motivational and cognitive variables with respect to the management of students’ emotional reactions. As a result, it is recommended that achievement motivation and creative self-efficacy should be regarded by educators as significant goals among instructional practice variables to improve the situation of creative anxiety.

## 6. Theoretical Contributions

This study makes the following theoretical contributions: 

(1) It enriches the literature on the antecedents of creative anxiety. While most previous studies have focused on individual characteristics, such as personality traits and emotional intelligence (Akpur, 2024; Hoffmann et al., 2021), this study explores the impacts of situational variables—namely, teachers’ innovative support behaviors—and clarifies the mechanisms through which students’ creative anxiety is influenced by external support. This study thus broadens the understanding of how contextual and interpersonal variables affect creative emotional experiences.

(2) It confirms the roles of creative self-efficacy and achievement motivation. This study not only validates their independent mediating effects but also explores their chain mediation pathway, thus providing clues about how motivation and cognition influence emotional responses. In this way, our understanding of the psychological processes underlying creative anxiety is improved.

(3) It enriches the theoretical framework of innovation education. This study incorporates Conservation of Resources Theory, Self-Determination Theory, and Social Cognitive Theory into a theoretical framework for art and design education, providing a theoretical basis and foundation for future empirical studies on creativity and emotional regulation in educational settings.

## 7. Practical Implications

This study focuses on eliminating anxiety in the field of creativity, as an aspect of emotional intelligence that impedes students’ creative potential in art and design education. The results revealed that the innovative support behaviors of teachers play a directly alleviating role, as well as a mediating role through significant psychological variables. Thus, this study provides a practical model for educational intervention.

First, instructors should create a learning context that offers psychological safety, adequate learning resources, and positive evaluation, representing basic forms of psychological support that directly decrease the perception of threat in the field of creativity. Second, educational practices should scaffold students’ creative self-efficacy by offering appropriately complex projects and acknowledging their incremental successes, which helps them to feel more confident in their creative ability. At the same time, stimulating achievement motivation through clear and challenging goals is expected to enable students to focus on problems, rather than anxiety.

The most important implication of this study is the integrated effect of the mediators. As achievement motivation stimulated by support not only decreases anxiety, but also increases self-efficacy (which further decreases anxiety), educators should provide holistic experiences for students by creating both motivation and confidence, thus taking advantage of these sequential effects.

## 8. Limitations and Future Directions

Although this study revealed several important findings, there are also several limitations. First, the cross-sectional design of the study limited the possibility of drawing causal conclusions regarding the sequential mediation effects. Future research could use multi-wave longitudinal designs or experimental methods to more rigorously test the causal and temporal relationships proposed through the model. Second, the reliance on self-reported questionnaires, which were all collected within the same period of time, may introduce common method bias. Although we conducted procedural controls and preliminary statistical tests, more robust statistical techniques—such as the Unmeasured Latent Method Construct (ULMC) approach—are recommended for future studies to better control for this potential bias. Third, the participants in the present study were recruited via a convenience sampling method from six universities in mainland China, which may constrain the generalizability of the findings. Future research could employ random or stratified sampling across a more diverse range of institutions and cultural contexts to enhance the external validity and allow for cross-cultural comparisons. Fourth, while theoretical relationships were examined, the analytical models did not control for potential confounding variables (e.g., gender, age). Future studies should incorporate relevant covariates to purify the observed relationships and strengthen the internal validity of the findings. Finally, the theoretical integration of Conservation of Resources Theory, Self-Determination Theory, and Social Cognitive Theory, while intended to provide a comprehensive framework, presents conceptual challenges. Future work should aim to develop a more cohesive theoretical model that explicitly reconciles the external, internal, and cognitive drivers of creative anxiety.

## 9. Conclusions

This study demonstrated that the innovative support behaviors of teachers have a significantly negative influence on creative anxiety in art and design majors; furthermore, the independent and the chained mediating influences of creative self-efficacy and achievement motivation in this relationship were verified. The findings are not only useful in extending the existing knowledge regarding creative anxiety, but also provide useful theoretical information and practical directions for novel learning activities. Through the creation of positive teaching conditions and placing emphasis on enhancing the levels of self-efficacy and achievement motivation among students, educators may be able to reduce the occurrence of anxiety associated with creative assignments, consequently enriching the creative potential of the students.

## Figures and Tables

**Table 1:** Means, standard deviations, and correlations for study variables (*N* = 785). *Note*: *** *p* < 0.001.

Variable	M	SD	1	2	3	4
1. TIS	3.85	0.72	1			
2. CSE	3.65	0.81	0.52 ***	1		
3. AM	3.91	0.68	0.48 ***	0.56 ***	1	
4. CA	3.28	0.85	−0.36 ***	−0.45 ***	−0.41 ***	1

**Table 2:** Results of the chain mediation analysis involving creative self-efficacy and achievement motivation (*N* = 785).

Dependent Variable	Independent Variable	R	R^2^	F	β	t
CA	TIS	0.631	0.398	255.756 ***	−0.631 (0.039)	−15.992 ***
CSE	TIS	0.811	0.657	740.762 ***	0.811 (0.030)	27.217 ***
AM	TIS	0.858	0.736	537.874 ***	0.485 (0.045)	10.866 ***
	CSE				0.416 (0.045)	9.322 ***
CA	TIS	0.664	0.441	101.372 ***	−0.285 (0.074)	−3.833 ***
	CSE				−0.241 (0.072)	−3.353 ***
	AM				−0.183 (0.074)	−2.466 *

Note: * *p* < 0.05; *** *p* < 0.001.

**Table 3:** Bootstrap results for the chain mediation effects.

Path	Effect	BootSE	BootLLCI	BootULCI
Total Effect	−0.631	0.039	−0.708	−0.553
Direct Effect	−0.285	0.074	−0.431	−0.139
Total Indirect Effect	−0.346	0.069	−0.481	−0.210
TIS → CSE → CA	−0.196	0.069	−0.329	−0.060
TIS → AM → CA	−0.089	0.041	−0.170	−0.012
TIS → CSE → AM → CA	−0.062	0.029	−0.121	−0.008

## Data Availability

The data that support the findings of this study are available from the corresponding author upon reasonable request.
